# Effect of water content on the glass transition temperature of mixtures of sugars, polymers, and penetrating cryoprotectants in physiological buffer

**DOI:** 10.1371/journal.pone.0190713

**Published:** 2018-01-05

**Authors:** Andrew C. Drake, Youngjoo Lee, Emma M. Burgess, Jens O. M. Karlsson, Ali Eroglu, Adam Z. Higgins

**Affiliations:** 1 School of Chemical, Biological and Environmental Engineering, Oregon State University, Corvallis, Oregon, United States of America; 2 Department of Mechanical Engineering, Villanova University, Villanova, Pennsylvania, United States of America; 3 Department of Neuroscience and Regenerative Medicine, Medical College of Georgia, Augusta University, Augusta, Georgia, United States of America; 4 Department of Obstetrics and Gynecology, Medical College of Georgia, Augusta University, Augusta, Georgia, United States of America; University of Maryland, UNITED STATES

## Abstract

Long-term storage of viable mammalian cells is important for applications ranging from *in vitro* fertilization to cell therapy. Cryopreservation is currently the most common approach, but storage in liquid nitrogen is relatively costly and the requirement for low temperatures during shipping is inconvenient. Desiccation is an alternative strategy with the potential to enable viable cell preservation at more convenient storage temperatures without the need for liquid nitrogen. To achieve stability during storage in the dried state it is necessary to remove enough water that the remaining matrix forms a non-crystalline glassy solid. Thus, the glass transition temperature is a key parameter for design of cell desiccation procedures. In this study, we have investigated the effects of moisture content on the glass transition temperature (*T*_*g*_) of mixtures of sugars (trehalose or raffinose), polymers (polyvinylpyrrolidone or Ficoll), penetrating cryoprotectants (ethylene glycol, propylene glycol, or dimethyl sulfoxide), and phosphate buffered saline (PBS) solutes. Aqueous solutions were dried to different moisture contents by equilibration with saturated salt solutions, or by baking at 95°C. The glass transition temperatures of the dehydrated samples were then measured by differential scanning calorimetry. As expected, *T*_*g*_ increased with decreasing moisture content. For example, in a desiccation medium containing 0.1 M trehalose in PBS, *T*_*g*_ ranged from about 360 K for a completely dry sample to about 220 K at a water mass fraction of 0.4. Addition of polymers to the solutions increased *T*_*g*_, while addition of penetrating cryoprotectants decreased *T*_*g*_. Our results provide insight into the relationship between relative humidity, moisture content and glass transition temperature for cell desiccation solutions containing sugars, polymers and penetrating cryoprotectants.

## Introduction

Maintaining the viability mammalian cells during long term storage is important in many areas, including embryo preservation for breeding of domestic animals [1], species conservation [2] and assisted human reproduction [3], as well as preservation of cell-based therapeutic products such as stem cells [4]. Currently, the leading method for long-term storage of mammalian cells is cryopreservation, which requires that cells be kept at temperatures near the boiling point of nitrogen (-196°C). The requirement for storage in liquid nitrogen increases costs and makes it more challenging to ship the cells from the storage facility to the end user. To address these drawbacks, several researchers have recently begun to investigate the potential for cell desiccation to enable storage at higher temperatures [5–11]. While results have been encouraging in some cases (e.g., blood platelets [12]), mammalian cells have generally proven to be difficult to preserve in the dry state.

In contrast to mammalian cells, several other organisms (including yeast, tardigrades and even larger organisms such as resurrection plants) can survive nearly complete dehydration [13]. These organisms have developed adaptations that provide protection at low water content, most notably the accumulation of sugars such as trehalose and sucrose [13]. The study of these organisms has resulted in a developing consensus about the physical mechanisms of desiccation protection. Sugars provide protection by directly interacting with polar groups on biomolecules such as lipids and proteins, stabilizing them at low water content. In essence, the sugars are thought to replace the hydrogen bonds that would normally be made by water. For this reason, this protective mechanism is known as the water replacement hypothesis [14]. Another key mechanism of desiccation protection is the formation of aqueous glass, which embeds cellular components in a noncrystalline solid matrix and slows down degradative chemical reactions [14].

Sugars such as trehalose and sucrose are commonly used in studies of mammalian cell desiccation [5–11]. For effective protection, sugars must be present on both sides of the biological membranes [15–18]. This presents a challenge for mammalian cells, which lack membrane transporters for sugars such as trehalose and sucrose. While various strategies have been devised for getting trehalose across the plasma membrane [16, 19–23], it remains a challenge to ensure distribution into intracellular organelles.

Penetrating cryoprotectant additives (CPAs), such as dimethyl sulfoxide (DMSO), ethylene glycol (EG), and propylene glycol (PG), are membrane permeable and may offer protection against dehydration stresses in the cytoplasm as well as within organelles. Such CPAs have been shown to mitigate damage from cellular dehydration during cryopreservation [24, 25]. When used in combination with intracellular sugars, small amounts of DMSO have been shown to enable successful cryopreservation of mouse oocytes [18, 26]. While penetrating CPAs are generally poor glass formers compared with sugars, they may interact with and stabilize membrane phospholipids and other biomolecules at low water content. Thus, the combination of sugars and penetrating CPAs may be useful for cell desiccation.

Polymers such as dextran and polyvinylpyrrolidone (PVP) have also been studied as additives during cell desiccation [27, 28]. Polymers are known to be good glass formers, but are relatively difficult to get into cells and generally do not form stabilizing interactions with biomolecules [14]. Thus, they are typically used in combination with a sugar such as trehalose to help promote formation of an extracellular glass.

One of the most important performance characteristics of a desiccation solution is its ability to form a glass as water is removed during drying. There have been numerous studies of the glass formation properties of binary aqueous solutions, especially the water-trehalose system [29]. However, glass transition data for more complex multi-component solutions is relatively scarce. The glass transition temperature (*T*_*g*_), which refers to a narrow temperature range in which the material undergoes a second-order phase transition (i.e., liquid to glass or vice versa), ultimately determines the appropriate biospecimen storage temperature. Consequently, different methods such as differential scanning calorimetry (DSC) and thermomechanical analysis have been developed to measure *T*_*g*_ [30]. DSC is the most widely used technique to determine *T*_*g*_ of biopreservation mixtures based on a change in the heat capacity [5, 14, 31]. In this study, we used DSC to examine the glass transition properties of aqueous solutions containing various combinations of sugars (trehalose or raffinose), polymers (PVP or Ficoll), penetrating CPAs (DMSO, EG, or PG), and phosphate buffered saline (PBS). The resulting data will be useful for selecting promising compositions for cell desiccation media.

## Materials and methods

### Sample preparation

Mixtures with the compositions shown in Table 1 were investigated. All aqueous solutions were prepared using purified water (ACS grade, Ricca Chemical Company, Arlington, TX) and filtered using a 0.45-μm filter. Trehalose dihydrate (≥ 99% purity) was obtained from Sigma Aldrich (St. Louis, MO). Raffinose (99% purity) was obtained from Alfa Aesar (Ward Hill, MA). Polyvinylpyrrolidone (99% purity, ~40 kDa) was obtained from Amresco (Solon, OH). Ficoll PM 70 (~70 kDa) was obtained from GE Healthcare (Pittsburg, PA). Dimethyl sulfoxide (≥ 99.9% purity) and ethylene glycol (≥ 99% purity) were obtained from Avantor Performance Materials (Center Valley, PA). Propylene glycol (≥ 99.5% purity) was obtained from VWR (Radnor, PA). Phosphate buffered saline was prepared by mixing 8 g of sodium chloride (EMD Millipore, Burlington, MA), 0.2 g of potassium chloride (Avantor Performance Materials), 2.16 g of sodium monohydrogen phosphate heptahydrate (VWR), and 0.2 g of potassium dihydrogen phosphate (Mallinckrodt Chemicals, St. Louis, MO) in purified water raised to a total volume of 1 L volume then corrected to pH 7.4 using ~1 mL of 1 M HCl. PBS containing calcium and magnesium (PBS Mg/Ca) was obtained from Quality Biological (Gaithersburg, MD).

**Table 1:** Mixture compositions for glass transition studies.

Composition of Desiccation Solution	Corresponding Solute Mass Percentages
0.1 M trehalose in water	trehalose (100%)
0.1 M trehalose in PBS ^a^	trehalose (78.2%), PBS salts (21.8%)
0.1M trehalose, 10% PVP in water	trehalose (25.5%), PVP (74.5%)
0.1 M trehalose, 10% Ficoll in PBS	trehalose (23.6%), PBS salts (7.3%), Ficoll (69.1%)
0.1 M trehalose, 10% PVP in PBS	trehalose (23.6%), PBS salts (7.3%), PVP (69.1%)
0.1 M trehalose, 10% PVP in PBS Mg/Ca ^b^	trehalose (23.6%), PBS Mg/Ca salts (7.3%) PVP (69.1%)
0.1 M raffinose, 10% PVP in PBS	raffinose (31.5%), PBS salts (6.0%), PVP (62.5%)
0.1 M trehalose, 10% PVP, 0.25 M DMSO in PBS	trehalose (20.8%), PBS salts (6.4%), PVP (60.9%), DMSO (11.9%)
0.1 M trehalose, 10% PVP, 0.25 M EG in PBS	trehalose (21.3%), PBS salts (6.6%), PVP (62.4%), EG (9.7%)
0.1 M trehalose, 10% PVP, 0.25 M PG in PBS	trehalose (20.9%), PBS salts (6.4%), PVP (61.1%), PG (11.6%)
0.1 M trehalose, 10% PVP, 0.5 M DMSO in PBS	trehalose (18.6%), PBS salts (5.8%), PVP (54.4%), DMSO (21.2%)
0.1 M trehalose, 10% PVP, 0.5 M EG in PBS	trehalose (19.5%), PBS salts (6%), PVP (56.9%), EG (17.6%)
0.1 M trehalose, 10% PVP, 0.5 M PG in PBS	trehalose (18.7%), PBS salts (5.8%), PVP (54.7%), PG (20.8%)

^a^ PBS is phosphate buffered saline without calcium and magnesium (see text for details).

^b^ PBS Mg/Ca is phosphate buffered saline with calcium and magnesium.

To prepare samples with different moisture contents, mixtures containing sugar (trehalose or raffinose), PBS salts, and polymers (PVP or Ficoll) in various combinations were equilibrated in defined relative humidity environments at room temperature (~21°C). Four different relative humidities, ranging from 6.5% to 75%, were established using saturated salt solutions sealed in mason jars (Table 2). Samples were placed on stacked 3D printed platforms (acrylonitrile butadiene styrene filament) within the mason jars, to hold the specimens above the saturated salt solutions. Two different approaches were used to prepare the samples to be placed in the mason jars. The first involved placing aqueous solution into the sample containers within the sealed mason jars, thus allowing equilibration of the samples by loss of water to the controlled relative humidity environment. These samples were weighed at 2-day intervals until the mass stabilized, which occurred after approximately 3 weeks. For the second approach, the samples were first lyophilized overnight in a benchtop freeze dryer with a condenser temperature of -56°C (Virtis Lyo-Centre: 3.5L DBT ES-55) and then placed in sample pans within the controlled relative humidity environments. In this case, samples typically equilibrated by absorbing moisture from the air. These samples were equilibrated for 2–3 weeks, until their mass had stabilized.

**Table 2:** Relative humidity values at room temperature for saturated salt solutions [32].

Salt Solution	Relative Humidity (%)
Lithium Bromide, LiBr	6.5
Potassium Acetate, KCH_3_COO	23
Magnesium Nitrate, Mg(NO_3_)_2_	54
Sodium Chloride, NaCl	75

Two different types of sample containers were used depending on the intended use of the sample. For moisture content determination, samples were equilibrated in mason jars within disposable aluminum pans (2.8 cm diameter, 1 cm depth, VWR). Samples destined for differential scanning calorimetry (DSC) studies were equilibrated in Tzero DSC sample pans (part number 901683.901, TA Instruments, New Castle, DE). Samples were prepared in triplicate and each replicate was equilibrated in a separate relative humidity chamber.

To prepare samples containing any of the penetrating CPAs, it was not possible to equilibrate mixtures in controlled relative humidity environments as described above. This is because these CPAs are volatile and can be lost from the sample during the equilibration process. Therefore, any mixtures containing DMSO, EG, or PG (see Table 1) were created by equilibrating a CPA-free mixture in mason jars to achieve a range of moisture contents, and then adding an appropriate volume of CPA to the sample to achieve the desired CPA content.

Completely dry samples for DSC experiments were prepared by placing the mixture of interest in a DSC pan and baking it in an oven at 95°C for 6–7 days. The DSC pan was then hermetically sealed and used in DSC experiments. By testing samples of 0.5 M trehalose-water that had dried for 3, 5, 6, 7, and 9 days, it was determined that 6–7 days were sufficient to dry the samples. After 6 days of drying, the glass transition temperature of samples stabilized, and remained stable after up to 9 days of drying (data not shown).

### Determination of the glass transition temperature

**Fig 1:**
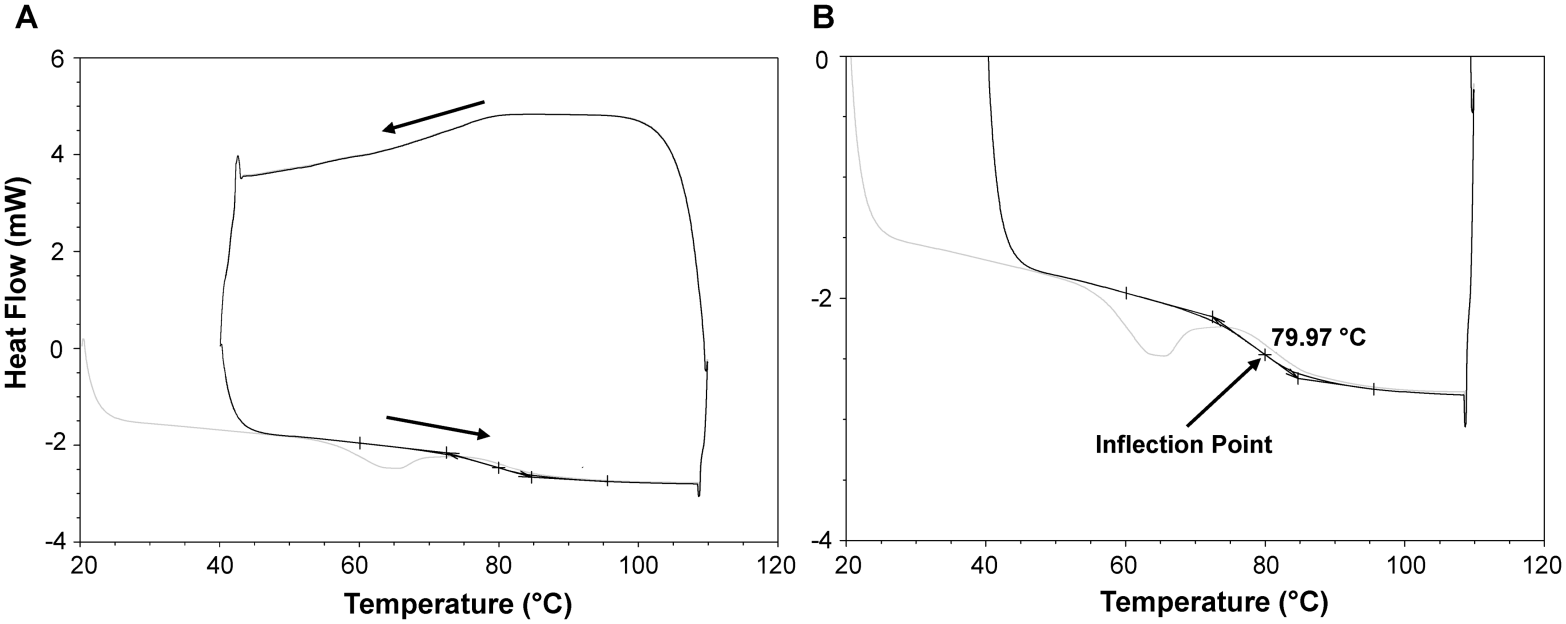
Representative DSC data (A) and zoomed in version showing the glass transition region (B). Light grey line is the initial ramp up in temperature which is done to erase thermal history in the sample. The test proceeds counter-clockwise, as indicated by the arrows. The glass transition temperature was determined from the inflection point in the second heating cycle, as illustrated in panel B (arrow).

After removing equilibrated DSC pans from the mason jars, the pans were immediately weighed and hermetically sealed with aluminum hermetic lids (part number 901684.901, TA Instruments), using a Tzero Sample Press (TA Instruments). The glass transition temperatures of the samples were then determined using a Q2000 DSC with an RCS90 cooling system (TA Instruments), following the ASTM standard test method [33]. Briefly, samples underwent two cycles in which they were heated at 10°C/min to a temperature 20–30°C higher than the expected glass transition temperature, and then cooled at 20°C/min to a temperature approximately 50°C lower than the expected glass transition temperature. The glass transition temperature was determined from the inflection point observed in the second heating cycle, using TA Universal Analysis software. Fig 1 is representative of a typical experiment. The DSC temperature and heat flow were calibrated using an indium standard. The accuracy of the temperature calibration was periodically checked by measuring the melting temperature of purified water (Ricca Chemical Company), resulting in a measured melting point within ±1 K of the expected value.

### Models for predicting the glass transition temperature

Various models have been used to predict glass transition temperatures of mixtures, as reviewed recently by Katkov and Levine [34] and Corti et al [35]. The most commonly used are the Gordon-Taylor (GT) model and the Couchman-Karasz (CK) model. The GT model allows prediction of the glass transition temperature of a mixture as a function of the glass transition temperatures of the pure components and a parameter *k* that characterizes the strength of the interaction between the two components [36]:

$$T_{g,mix}=(w_{2}T_{g2}+kw_{1}T_{g1})/(w_{2}+kw_{1})$$ (1)

where *w* is the mass fraction and the subscripts 1 and 2 refer to water and solute, respectively. While the GT model is typically used for binary solutions, we use it here to characterize more complicated solutions by treating all non-water components as a single “lumped” component, as has been described previously [37]. The GT model was fit to experimental data using a least squares approach.

The CK model allows prediction of *T*_*g*,*mix*_ for a mixture given the glass transition temperature (*T*_*g*,*i*_) and the corresponding change in heat capacity (Δ*C*_*p*.*i*_) for the *i*-th pure component [38]:

$$T_{g,mix}=\text{exp}\left(\frac{\sum_{i}^{n}w_{i}\Delta C_{p,i}\text{ln}T_{g,i}}{\sum_{i}^{n}w_{i}\Delta C_{p,i}}\right)$$ (2)

where *n* is the number of components in the mixture.

### Determination of moisture content

All samples used for the determination of moisture content were equilibrated in chambers with defined relative humidity environments shown in Table 2. For trehalose-water samples, additional saturated-salt relative humidity chambers were also used, namely LiCl (11% relative humidity), and MgCl_2_ (33% relative humidity). Those relative humidity chambers were not used for the other sample compositions, due to space constraints. After equilibration of samples in the mason jars, the aluminum pans were weighed and placed into an oven at 95°C to remove any residual water. Samples were removed from the oven after 7 days of drying, and weighed again. This dry mass was compared to the initial sample mass to determine the mass fraction of water in the original sample, as well as the residual moisture content (g water/g dry mass). The resulting moisture content data were fit using the Brunauer, Emmett and Teller (BET) model [39]:

$$m=\frac{m_{m}ca_{w}}{\left(1-a_{w})(1+(c-1)a_{w}\right)}$$ (3)

where *m* is the residual moisture content, *a*_*w*_ is the water activity, and *m*_*m*_ and *c* are constants. To obtain best-fit values of *m*_*m*_ and *c*, Eq 3 was linearized [40] and fit to the data using linear regression.

## Results and discussion

### Moisture sorption isotherms

**Fig 2:**
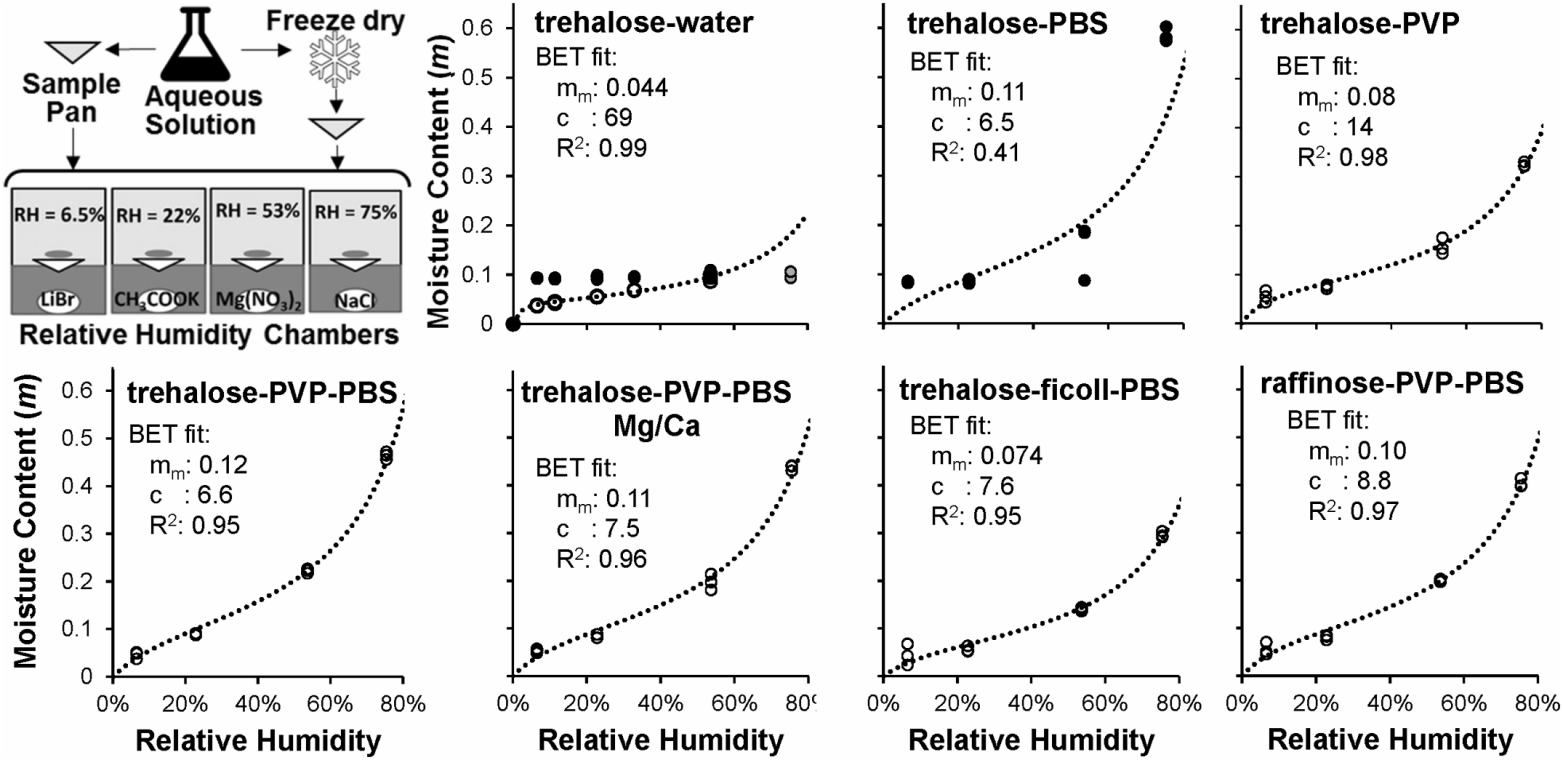
Moisture sorption isotherms. Moisture content in samples prepared either as an aqueous solution (black symbols) or as a freeze-dried matrix (white and grey symbols) was measured after equilibration in chambers with controlled relative humidity. The BET model (Eq 3) was fit (dotted lines) to observations from freeze-dried samples, except for the trehalose-PBS case, in which aqueous solution data were used for fitting; in the trehalose-water experiment, outlier values at 75% relative humidity (grey symbols) were omitted from the fit. See Table 1 for detailed composition of each mixture.

Dry basis moisture content values for various mixtures are shown in Fig 2 as a function of relative humidity. When binary trehalose-water mixtures were dried from an aqueous solution, the moisture content observed after equilibration was about 0.104 at a relative humidity of 54% and dropped slightly to 0.095 at a relative humidity of 33%. However, further decreases in relative humidity did not decrease the moisture content, suggesting that the samples may not have achieved equilibrium for relative humidities below 33%. In contrast, when aqueous trehalose solutions were freeze-dried before equilibration in controlled relative humidity environments, much lower moisture contents were observed in the samples. For example, as shown in Fig 2, the moisture content for a relative humidity of 6.5% was 0.037 for the freeze-dried sample, much lower than the value of 0.093 that was obtained when the sample was prepared by directly drying an aqueous solution. The BET fit to the data for freeze-dried trehalose is excellent over the relative humidity range 6.5%-54%, but substantially overestimates the moisture content for a relative humidity of 75%. The uncharacteristically low moisture content of 0.10 observed at 75% relative humidity may be a result of crystallization of trehalose dihydrate, as has been suggested previously [41]. Overall, our moisture content results for freeze-dried trehalose-water mixtures are very similar to those in previous reports [41, 42].

Trehalose-PBS mixtures repeatedly failed to produce a dry sample after freeze-drying. This may have been caused, at least in part, by sample inhomogeneity due to collapse of the matrix during freeze-drying. Thus, for trehalose-PBS mixtures, samples were equilibrated from aqueous solution and not from the freeze-dried matrix. In this case, the samples were allowed to equilibrate for 3 months. As shown in Fig 2, trehalose-PBS mixtures yielded moisture content values that remained below 0.18 for relative humidities up to 54%, but increased sharply to 0.60 when the relative humidity increased to 75%. The data are somewhat scattered for the trehalose-PBS mixtures, and the BET fit to the data is poor, especially at the highest relative humidity value. Nonetheless, the results are approximately consistent with published results for mixtures of trehalose, PBS and water [31].

Similar problems with cake collapse during freeze drying were not observed for the other mixtures. The moisture sorption isotherms for all mixtures containing PVP were similar, yielding moisture contents ranging from about 0.04 to 0.47 over the range of relative humidities tested (see Fig 2). This is consistent with previously published results for binary PVP-trehalose mixtures [43]. Samples with Ficoll retained less moisture than did PVP mixtures, yielding moisture contents ranging from 0.02 to 0.30.

### Glass transition temperatures

#### Trehalose-water mixtures

**Fig 3:**
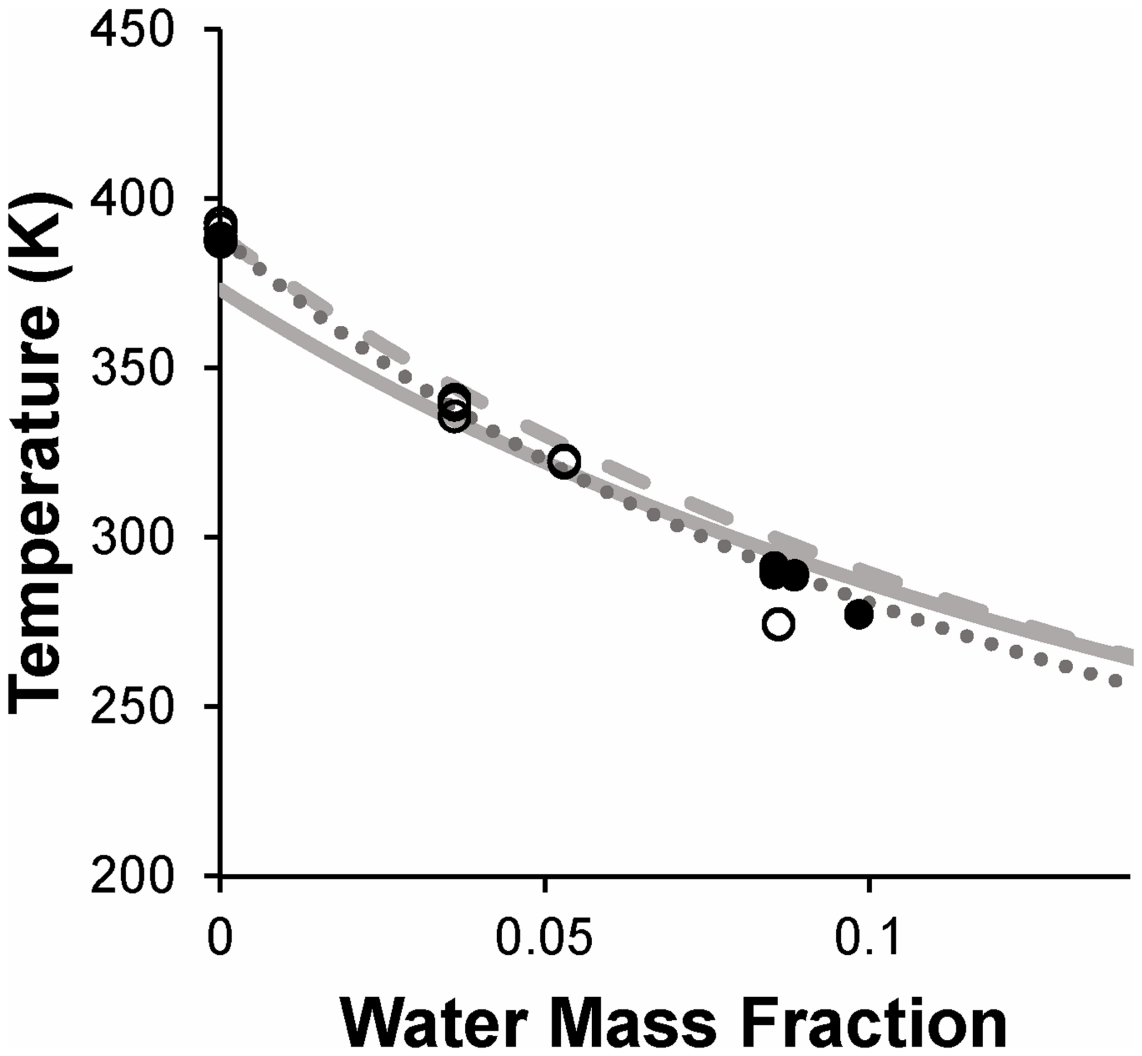
Glass transition temperature of binary trehalose-water mixtures. Samples were prepared either by equilibrating aqueous solution (black symbols) or freeze-dried matrix (white symbols) in various relative humidity environments. The solid grey line shows a fit of the GT model to literature data, as reported by Chen et al. [29]. The dotted line shows the best-fit GT model (Eq 1) to the data from the current study. The dashed line represents predictions of the CK model (Eq 2) using the parameters shown in Table 3.

Fig 3 shows glass transition temperatures obtained for binary mixtures of trehalose and water. Pure trehalose yielded an average glass transition temperature (*T*_*g*_) of 389 K; the eight replicates had *T*_*g*_ values that ranged from 387 K to 393 K. Literature values for the glass transition temperature of pure trehalose vary widely (reviewed in [29]), but several recent reports place *T*_*g*_ between 386 K and 394 K [43–47], consistent with our result. As expected, the glass transition temperature of trehalose-water mixtures decreased substantially as the water mass fraction increased, reaching about 280 K at 10% water content; this represents a decrease in *T*_*g*_ by more than 100 K. Chen and colleagues [29] compiled *T*_*g*_ data for trehalose-water mixtures from over 20 sources and fit the data using a GT model. As shown in Fig 3, our *T*_*g*_ data are reasonably consistent with the GT model of Chen et al. [29]. By fitting the GT model (Eq 1) to our own experimental results, we estimated a value *k* = 6.8 for the interaction parameter. Our estimate of the GT interaction parameter is higher than the value reported by Chen et al. (*k* = 5.2) [29] but slightly lower than the *k*-value reported by Crowe et al. (*k* = 7.5) [14]. Fig 3 also shows CK model (Eq 2) predictions based on published thermophysical properties of water and trehalose (Table 3). Overall, the results shown in Fig 3 demonstrate that our *T*_*g*_ results for trehalose-water mixtures are consistent with those reported in previous studies.

**Table 3:** Parameters for use in the CK model.

Species	*T*_*g*_ (K)	Δ*C*_*p*_ (J/g/K)	Sources
Water	136	1.94	Katkov and Levine, 2004 [34]
Trehalose	390	0.55	Katkov and Levine, 2004; Weng et al., 2014; Ohtake et al., 2004; Surana et al., 2004 [34, 45–47]
PVP (40,000 Da)	413	0.27	Buera et al., 1992 [48]

#### Trehalose-PVP-water mixtures

**Fig 4:**
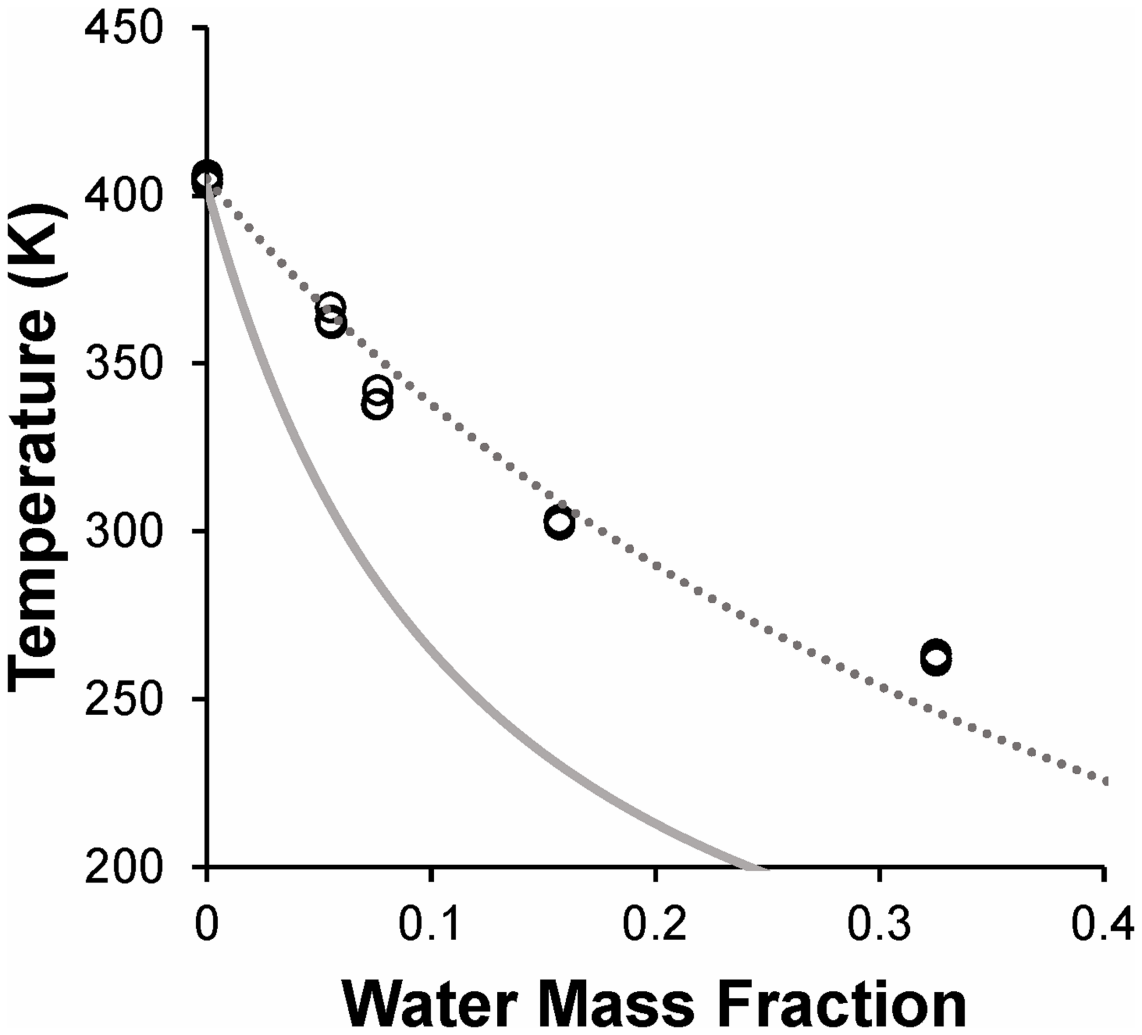
Glass transition temperature of trehalose-PVP-water mixtures. Samples were prepared by equilibrating freeze-dried matrix in various relative humidity environments. The dotted line shows the best fit of the GT model (Eq 1) to the data. The solid line shows predictions of the CK model (Eq 2) using the parameters shown in Table 3.

Fig 4 depicts the glass transition temperature as a function of moisture content for the ternary system trehalose-PVP-water. Compared to binary trehalose-water mixtures (Fig 3), the addition of PVP was found to increase the glass transition temperature. The glass transition temperature of dry trehalose-PVP (*T*_*g*_ = 405 K) was about 16 K higher than the *T*_*g*_ for dry trehalose, and *T*_*g*_ values were up to 30 K higher in the presence of PVP, over the full range of moisture contents tested. To our knowledge, there are no published studies of the glass transition properties of trehalose-PVP mixtures for PVP of the same molecular weight (40 kDa) as in the current study. Zhang and Zografi used PVP of a higher molecular weight, and reported a glass transition temperature of about 420 K for dry PVP-trehalose at the same mass ratio of trehalose to PVP as that used in our experiments [43]. This slightly higher *T*_*g*_ value is consistent with the expected increase in *T*_*g*_ with PVP molecular weight [48].

As shown in Fig 4, the GT model (Eq 1) yields an adequate fit to the data. In contrast, predictions of the CK model (Eq 2) were found to deviate greatly from the experimental observations for this ternary system, increasingly underestimating *T*_*g*_ as moisture content increased. The CK model assumes that intermolecular interactions between the components of the mixture are negligible. Because the CK model fails to predict *T*_*g*_ for trehalose-PVP-water mixtures, it is likely that the components do not behave in a manner consistent with the model assumptions. Previous studies of PVP-water mixtures have also shown large discrepancies between experimental data and the predictions of the CK model [48]. As a result, we did not attempt to use the CK model for analysis of the other sugar-polymer mixtures investigated in our study. The failure of the CK model is unfortunate, because the CK model would have allowed prediction of the glass transition point for mixtures of arbitrary composition, using only measurements made on the individual components, whereas the GT model requires experimental measurement of *T*_*g*_ for each mixture of interest.

#### Trehalose-PBS mixtures

**Fig 5:**
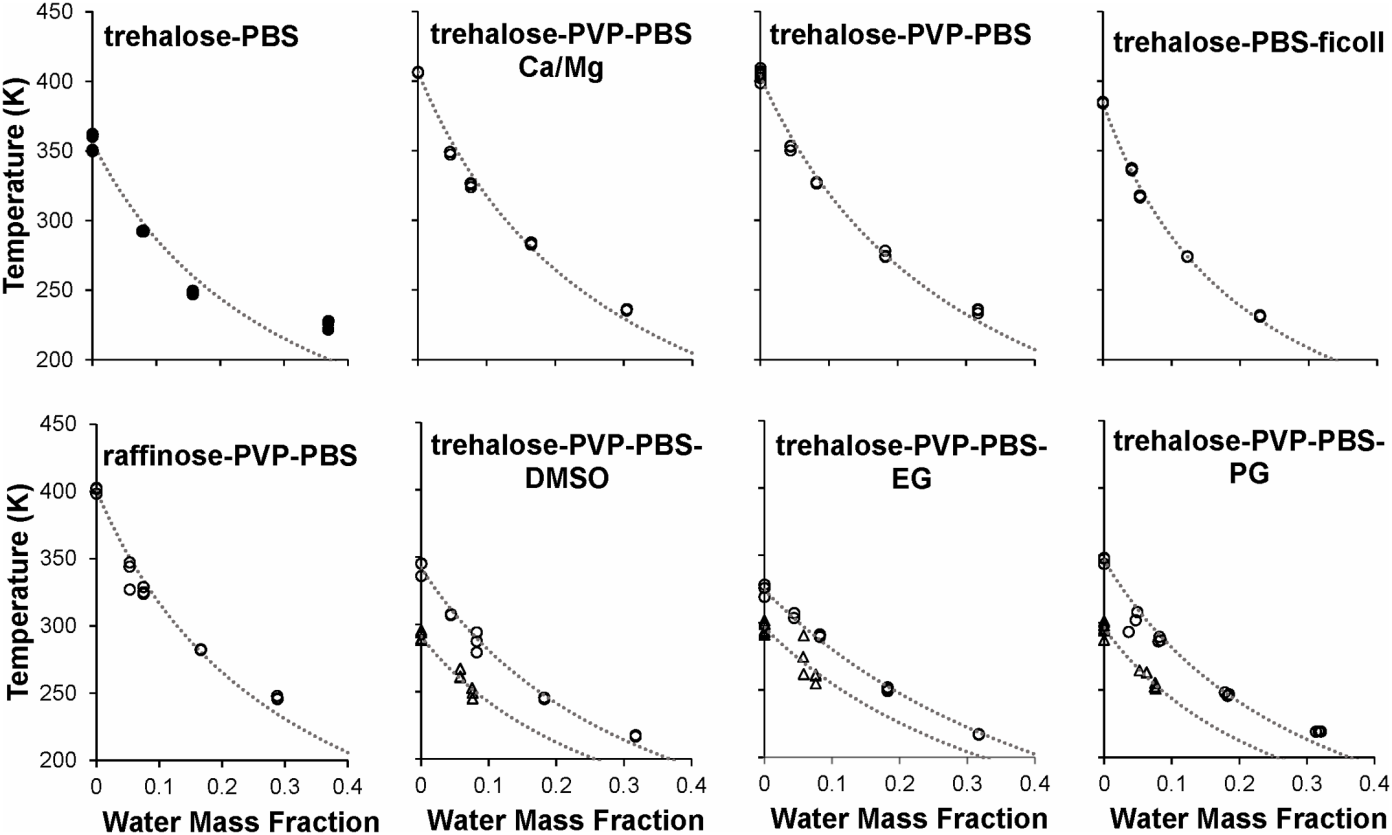
Glass transition temperatures of various mixtures containing PBS. Samples were prepared either by equilibrating aqueous solution (filled symbols) or freeze-dried matrix (open symbols) in various relative humidity environments. The dotted lines show the best-fit GT model to each data set. Mixtures containing penetrating CPAs at concentrations of 0.25 M and 0.5 M are shown as circles and triangles, respectively. See Table 1 for detailed composition of each mixture.

The glass transition temperatures for mixtures of trehalose and PBS are shown in Fig 5 as a function of water content. Compared with binary trehalose-water mixtures, the trehalose-PBS mixtures had a substantially lower glass transition temperature at low water content, which suggests that PBS salts impair the glass forming ability of trehalose. This result is consistent with other studies on the effects of PBS and similar physiological buffers on the glass transition temperature of trehalose mixtures [31, 37, 44, 46].

#### Sugar-PBS mixtures containing PVP or Ficoll

Compared with a baseline desiccation medium containing trehalose and PBS, mixtures that also include a polymer component had a higher glass transition temperature (Fig 5). In particular, the addition of PVP to trehalose-PBS increased *T*_*g*_ by about 40 K over the entire range of water mass fractions tested. Addition of Ficoll increased *T*_*g*_ to a lesser extent, resulting in *T*_*g*_ values about 30 K higher than those observed in the baseline trehalose-PBS desiccation medium. The presence of calcium and magnesium in the PBS did not have an appreciable effect on the glass transition temperature of trehalose-PVP-PBS mixtures. Replacement of trehalose with raffinose resulted *T*_*g*_ values that were similar to those obtained for trehalose-PVP-PBS mixtures. The dominant influence of PVP on *T*_*g*_ is most likely due to its high mass fraction relative to the other components, which may explain the apparent insensitivity of the glass transition temperature to variations in the buffer and sugar composition.

#### Trehalose-PBS-PVP mixtures containing penetrating CPAs

Fig 5 also depicts the effects of penetrating CPAs on the glass transition temperature of desiccation solutions. Compared to a baseline mixture containing trehalose, PVP and PBS without CPA, mixtures that also include a penetrating cryoprotectant additive (DMSO, EG, or PG) had a lower glass transition temperature. This decrease in *T*_*g*_ for the mixture is consistent with the lower *T*_*g*_ of each individual CPA compared with the glass transition points of pure trehalose and pure PVP. Interestingly, each of the three CPAs investigated had a similar effect; for dry mixtures, *T*_*g*_ decreased by nearly 55 K upon addition of 0.25 M CPA, and by nearly 110 K upon addition of 0.5 M CPA. In general, the drop in *T*_*g*_ as water content increased was less pronounced for mixtures containing a penetrating CPA than for mixtures without such additives.

#### Comparison of mixtures

**Fig 6:**
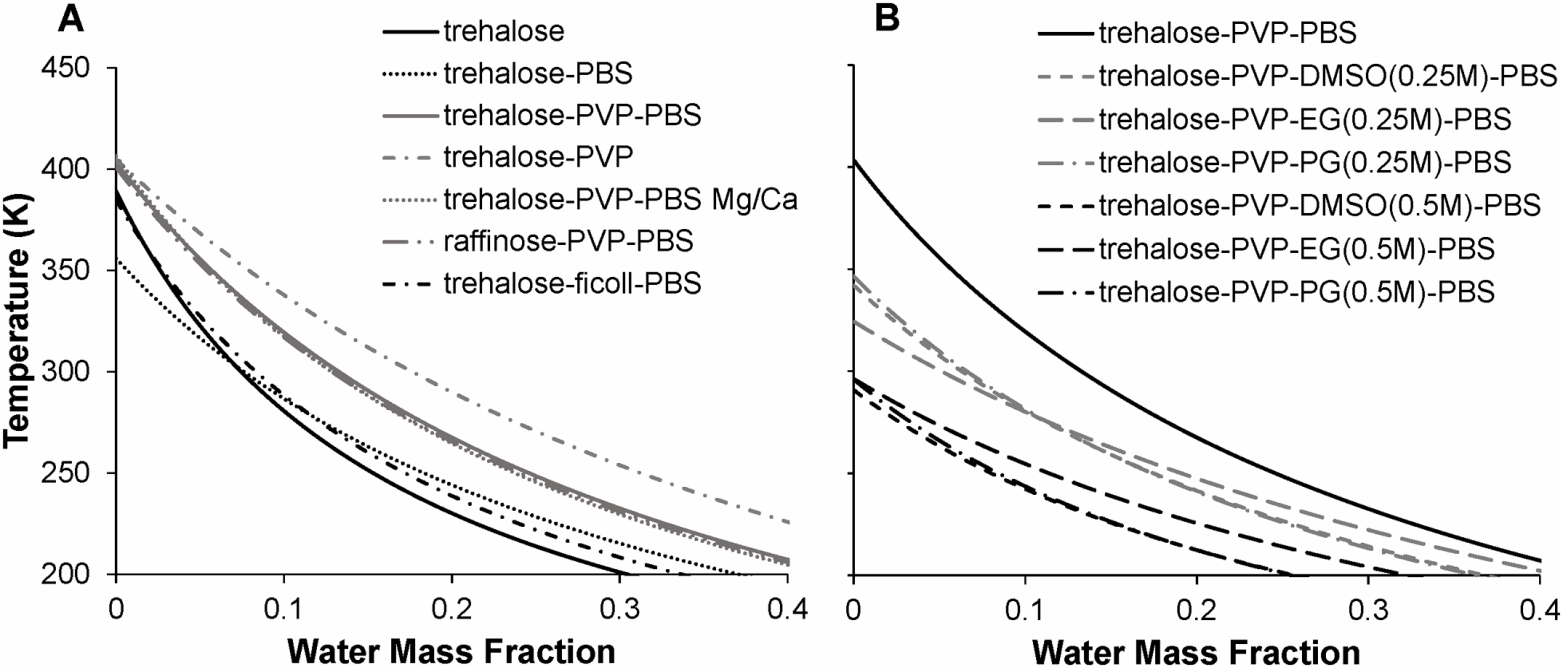
Comparison of best-fit GT models. (A) Effect sugars, polymers and PBS salts on the glass transition temperature. (B) Effect of CPAs on the glass transition temperature.

To facilitate comparison of the multi-component mixtures, the best-fit GT model for each mixture is plotted in Fig 6. The left panel of Fig 6 depicts the effects of sugars (trehalose and raffinose), polymers (PVP and Ficoll) and PBS salts on the glass transition temperature, whereas the right panel depicts the effects of penetrating CPAs (DMSO, EG, and PG) at two concentrations. The corresponding best-fit GT model parameters are summarized in Table 4. Several trends in the glass transition data are apparent in Fig 6:

- Compared with trehalose-water, trehalose-PBS has a lower *T*_*g*_, particularly at low water content (differences become less pronounced as water content increases);
- Addition of the polymers PVP or Ficoll to trehalose-PBS increases *T*_*g*_;
- PVP raises *T*_*g*_ to a greater extent than does Ficoll;
- Addition of any of the penetrating cryoprotectants DMSO, EG or PG to trehalose-PBS-PVP substantially decreases *T*_*g*_;
- Addition of calcium and magnesium to PBS does not have a discernible effect on the *T*_*g*_ of trehalose-PVP-PBS mixtures;
- Sugar-PVP-PBS mixtures yield similar *T*_*g*_ values whether the sugar component is trehalose or raffinose.

**Table 4:** Best-fit GT model parameters (see Eq 1).

Composition of desiccation solution	*T*_g2_ (K)	*k*
0.1 M trehalose in water	389	6.8
0.1 M trehalose in PBS	356	4.1
0.1M trehalose, 10% PVP	405	3.0
0.1 M trehalose, 10% Ficoll in PBS	385	5.7
0.1 M trehalose, 10% PVP in PBS	403	4.1
0.1 M trehalose, 10% PVP in PBS Mg/Ca	407	4.4
0.1 M raffinose, 10% PVP in PBS	401	4.2
0.1 M trehalose, 10% PVP, 0.25 M DMSO in PBS	342	3.8
0.1 M trehalose, 10% PVP, 0.25 M EG in PBS	324	2.8
0.1 M trehalose, 10% PVP, 0.25 M PG in PBS	347	4.0
0.1 M trehalose, 10% PVP, 0.5 M DMSO in PBS	291	4.1
0.1 M trehalose, 10% PVP, 0.5 M EG in PBS	296	3.2
0.1 M trehalose, 10% PVP, 0.5 M PG in PBS	296	4.4

## Conclusion

To our knowledge, this study provides the first measurements of the glass transition temperature in cell desiccation media containing a mixture of sugars, polymers and penetrating CPAs in physiological buffer. In general, the polymers PVP and Ficoll had a stabilizing effect; the addition of these polymers to a baseline desiccation media containing trehalose and PBS increased *T*_*g*_ substantially at any given moisture content. On the other hand, the addition of DMSO, EG or PG to the desiccation mixture decreased *T*_*g*_ in a concentration-dependent manner.

Our results provide a means for estimating the extent of drying necessary to achieve a glass transition temperature sufficiently high for stability during storage. For stable storage in the dried state, it has been suggested that *T*_*g*_ should be about 50 K above the storage temperature [49]. Thus, our results indicate that a desiccation solution containing 0.1 M trehalose and 10% PVP in PBS would need to be dried to a water mass fraction of about 5% for stable storage at room temperature. For storage in a refrigerator, less extensive drying to ~9% water content would be sufficient for stability. Addition of membrane-permeable CPAs to this desiccation mixture offers the potential for protection of intracellular organelles, but increases the extent of drying necessary for stable storage. Our results suggest that room-temperature storage of a desiccation solution containing 0.25 M CPA is not feasible, even after complete drying of the sample. However, it appears to be possible for such a solution to achieve stable storage in a standard refrigerator or freezer after drying to water mass fractions of ~3% and ~6%, respectively. These examples illustrate the potential of our results to facilitate design of promising multi-component mixtures for cell desiccation.

## Supporting information

S1 Glass Transition Data Glass transition temperatures for all mixtures. (XLSX)

S1 Moisture Sorption Data Moisture content data for all mixtures and relative humidity environments. (XLSX)
